# Incorporating Human Movement Behavior into the Analysis of Spatially Distributed Infrastructure

**DOI:** 10.1371/journal.pone.0147216

**Published:** 2016-01-19

**Authors:** Lihua Wu, Henry Leung, Hao Jiang, Hong Zheng, Li Ma

**Affiliations:** 1 Wuhan University, Department of Communication Engineering, Wuhan, 430072, China; 2 University of Calgary, Department of Electrical and Computer Engineering, Calgary, T2N 1N4, Canada; 3 Wuhan University, Geospatial Information Technology Cooperative Innovation Center, Wuhan, 430072, China; Beihang University, CHINA

## Abstract

For the first time in human history, the majority of the world's population resides in urban areas. Therefore, city managers are faced with new challenges related to the efficiency, equity and quality of the supply of resources, such as water, food and energy. Infrastructure in a city can be viewed as service points providing resources. These service points function together as a spatially collaborative system to serve an increasing population. To study the spatial collaboration among service points, we propose a shared network according to human's collective movement and resource usage based on data usage detail records (UDRs) from the cellular network in a city in western China. This network is shown to be not scale-free, but exhibits an interesting triangular property governed by two types of nodes with very different link patterns. Surprisingly, this feature is consistent with the urban-rural dualistic context of the city. Another feature of the shared network is that it consists of several spatially separated communities that characterize local people's active zones but do not completely overlap with administrative areas. According to these features, we propose the incorporation of human movement into infrastructure classification. The presence of well-defined spatially separated clusters confirms the effectiveness of this approach. In this paper, our findings reveal the spatial structure inside a city, and the proposed approach provides a new perspective on integrating human movement into the study of a spatially distributed system.

## Introduction

Migration is a phenomenon common to not only human but also animal populations. Millions of birds and insects undertake regular movement every year to cope with seasonal and environmental changes. Animals living in nature have many reasons to engage in these seasonal movements, ranging from the avoidance of food shortage [1] and the quest for physiologically optimal climates [2] to the avoidance of predation during periods of reproduction [3]. However, natural cycles also provide various species with sufficient resources in both permanent and temporary habitats, as well as the areas located between them, to meet energy requirements [4]. Uncovering how these spatially distributed service points cooperate to support migrating species is important for furthering our understanding of the ecosystem.

In our cities, there is a similar resource supply process. Each day, millions of people living in cities move between their workplace and home. Department stores, parking lots, gas stations and many other infrastructures can be viewed as service points of our resource supply system. Urban areas, which are inhabited by more than 50% of the global population, are experiencing unprecedented growth and intense competition. Thus, cities are emerging as key sites of social problem solving in the 21st century [5–7]. High population density in urban areas is accompanied by numerous challenges that city managers must address. The efficiency, equity and quality of the city resource supply are becoming key factors in the promise of urban sustainable development. To cope with these challenges, scientists and engineers worldwide have placed their hope on the smart city concept. In this new vision of the urban future, the city can be approached as a complex network in which infrastructures providing water, energy or other city services serve as interconnected components. These service points are sources of substantial amounts of data and provide dynamic insights into a society. By understanding a city through the lens of these service points, we gain new perspectives on the progress of city monitoring, measurement and management [8, 9].

As an important public infrastructure, the cellular network not only provides wireless communication services but also functions as an important data source that records human activities with the capability of localization. Since the discovery of the scaling law of human travel [10], call detail records (CDRs) from cellular networks have been widely used in a variety of research fields, such as human activity spaces and mobility patterns [11], epidemic control [12–14], traffic management [15,16] and localized recommendations [17]. Recently, greater attention has been paid to spatially embedded community detection [18], the extraction of geographic functions [19] and the attainment of spatial structures [20,21]. These studies fail to investigate potential applications of the data created by this type of infrastructure to improve its management or to understand resource supply mechanisms more generally, because CDR could not quantify communication resource usage. In cellular networks, base stations act as service points. They are distributed within cities and operate collaboratively to supply people with wireless communication resources. To better understand resource supply along the spatial structure and how a city operates, it is important to uncover collaboration patterns. Moreover, because people move in and out of service points, resource consumption at a single service point exhibits complicated changes and may fail to distinguish different group of service points. Considering the limited scope of human movement, service points that are close to each other will have a strong collaboration. Thus, service points may be classified into spatially separated clusters according to the collaboration relationship determined by people's resource consumption behavior. This classification will result in the more effective management of these service points.

To the best of our knowledge, this paper is the first to explore the spatial collaboration within a cellular network as a means of investigating a city’s resource supply. Furthermore, the study also utilizes data usage detail records (UDRs) as a secondary dataset. In contrast to CDR data, which focus more on human mobile calling behaviors, a UDR dataset contains more information about human movement patterns, with common data usage behaviors informing the high density location sampling, and quantifies communication resource consumption according to the traffic load. These data are more suitable for use in studies on spatial collaboration of resource supply. In this research, our data were collected in a city in western China (see Methods). Based on these UDRs of people's wireless resource usage behavior, we construct a shared network (see Methods) to study the spatially distributed resource supply system. This network contains two types of nodes with different link patterns. Surprisingly, these patterens are consistent with the urban-rural dualistic context of the city. In addition, it consists of several spatially separated communities determined by local people's active zones. According to these observations based on complex network [22] analysis, we propose a classification strategy to more effectively manage spatially distributed infrastructure.

## Results

### Two Types of Nodes with Different Link Patterns

**Fig 1:**
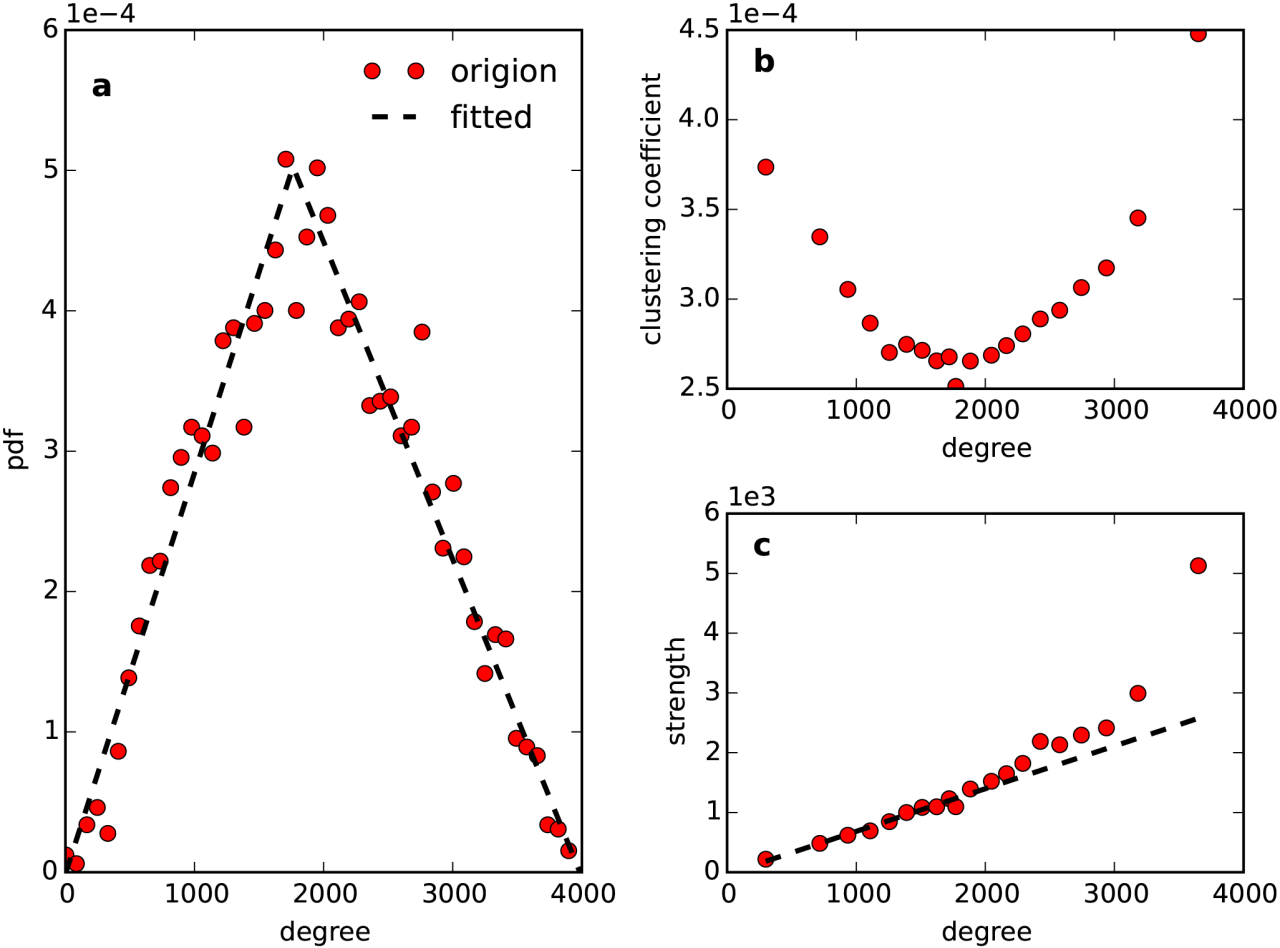
Two types of nodes. (a) is the pdf of the degree. The two dashed lines show the two categories of nodes. (b) shows the clustering coefficient versus degree. (c) shows strength versus degree. For low-degree nodes, we obtain *s*(*k*) ~ *αk*, where *s*(*k*) and *k* are the strength and the degree, respectively. Furthermore, *α* = 1.03±0.03 is approach to the average weight of the whole network, which is <*w*> = 0.95.

The degree distribution of the shared network is plotted in Fig 1a. It does not show any scale-free behavior; rather, degree distribution exhibits a triangular property. Fitting a triangular function in Eq (1) to the degree distribution, we obtain *l* = 4, *r* = 3984 and *m* = 1760. This triangular property indicates that the shared network consists of two categories of nodes. One type of nodes connects with many of the other nodes. We refer to these nodes as high-degree nodes because they have a high degree due to their large number of neighbors. The other type of nodes appears to connect with a fewer number of other nodes, and we call these nodes low-degree nodes accordingly.

$$P\left(k_{i}\right)=\{\begin{array}{ccc} \frac{2\left(k_{i}-l\right)}{\left(r-l\right)\left(m-l\right)} & l\leq k_{i}\leq m, & \\ \frac{2\left(m-k_{i}\right)}{\begin{array}{l} \left(r-l\right)\left(r-m\right) \end{array}} & m<k_{i}\leq r, & \\ 0 & otherwise. & \end{array}$$ (1)

These two types of nodes have a different local structure in the shared network. The clustering coefficient and strength are important metrics for studying the network structure. The clustering coefficient describes the intensity of the local structure [23], and the strength indicates the interdependence between weights and topology in a weighted network [24, 25]. If weights are independent of topology, we can find a linear relationship between the strength and the degree. Otherwise, the relationship between the strength and degree is nonlinear. As shown in Fig 1b and 1c, for low-degree nodes, the clustering coefficient decreases as the degree increases, and there is a linear relationship between the strength and the degree. By contrast, for high-degree nodes, the clustering coefficient increases as the degree increases, and the relationship between the strength and the degree is nonlinear. This result suggests that a low-degree node's edge weight is independent from its local topology; while for a high-degree node, as its number of neighbors increases, it becomes more likely to connect with a strong edge. Furthermore, as the number of neighbors grows, the local structure of a high-degree node becomes more intense, while the opposite occurs for that of a low-degree node.

**Fig 2:**
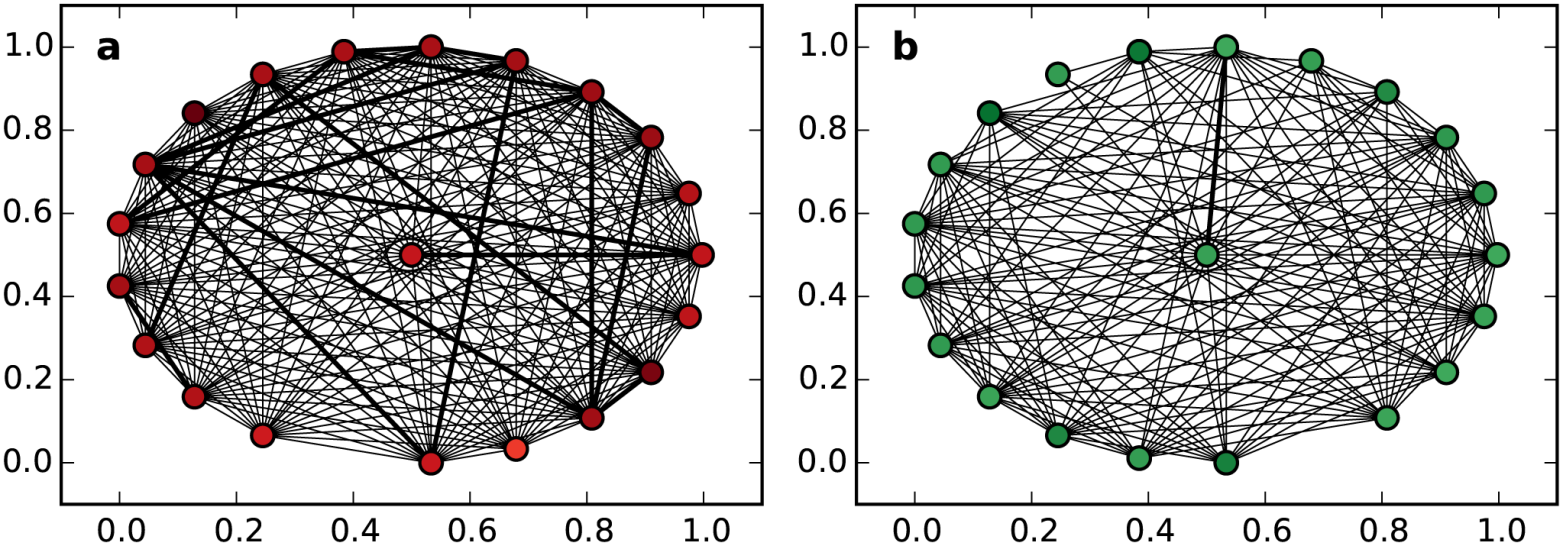
Neighbor connections. (a) shows the connections between high-degree nodes. (b) shows the connections between low-degree nodes. The width of edges is governed by the weight in the shared network. To simplify the visualization, we plot only the object node’s 20 closest neighbors, and we drop weak edges whose weight is smaller than 0.1.

To study the detailed link pattern, we plot the neighborhood of both types of nodes (see Fig 2). For a high-degree node, neighbors perform as a fully connected sub-network, with numerous connections between them. Many strong connections are found in this neighborhood. However, for a low-degree node, there are only a few weak connections in its neighborhood. Considering the performance of the clustering coefficient in Fig 1b, we can derive the change in the connections in the neighborhood from its neighbors’ growth. That is, for a high-degree node, as the local structure becomes more intense, the connections in its neighborhood will become stronger and more crowded; while for a low-degree node, connections between neighbors will become weaker or less crowded.

### Spatial Properties in the Shared Network

The above analysis raises some interesting questions about the network structure. Why do spatially distributed service points perform as different types nodes? How could these two types of nodes reach the same level of local structure intensity with nearly equal clustering coefficients? And why is the interdependence of weights and topology different for these two types of nodes? As the edge in the shared network exists only between service points providing resources to the same group of people, the degree of the node—which determines the node type—is affected by people’s spatial distribution and movement. To address these questions, we analyze the geographical space.

**Fig 3:**
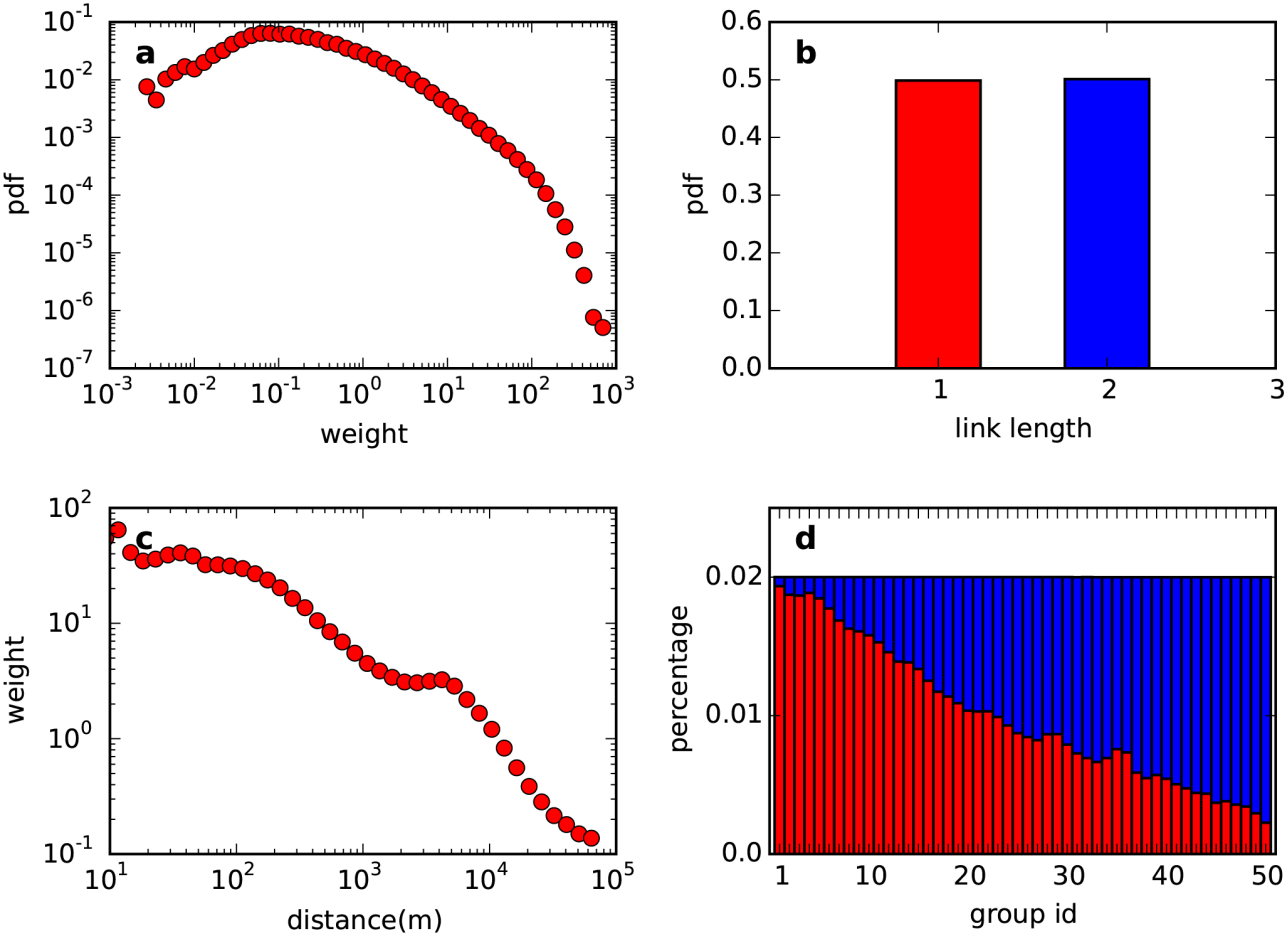
Edge weight and link length. (a) shows the distribution of weights. (b) shows the distribution of link lengths. (c) shows the change of weights as spatial distance increases. (d) shows the influence of spatial distance on the link length. Because there are only two different lengths of links, we distinguish them with red and blue colors.

Spatial distance clearly influences the network structure. Weights in the shared network follow a long tail distribution (see Fig 3a). Furthermore, there are two different lengths of links: 50% of the link lengths are equal to 1, and the other 50% are equal to 2. (see Fig 3b). Both of these metrics characterizing the relationship between node pairs change according to spatial distance. As shown in Fig 3c, weights decrease as spatial distance increases. In Fig 3d, we sort node pairs into ascending distant order and group them into 50 equal parts. A shorter length of links dominates the first several groups. However, as the distance increases, a longer length of links gradually begins to dominate. This figure indicates the basic distance decay effect in the shared network, that is, spatial distance weakens the connection between service points.

**Fig 4:**
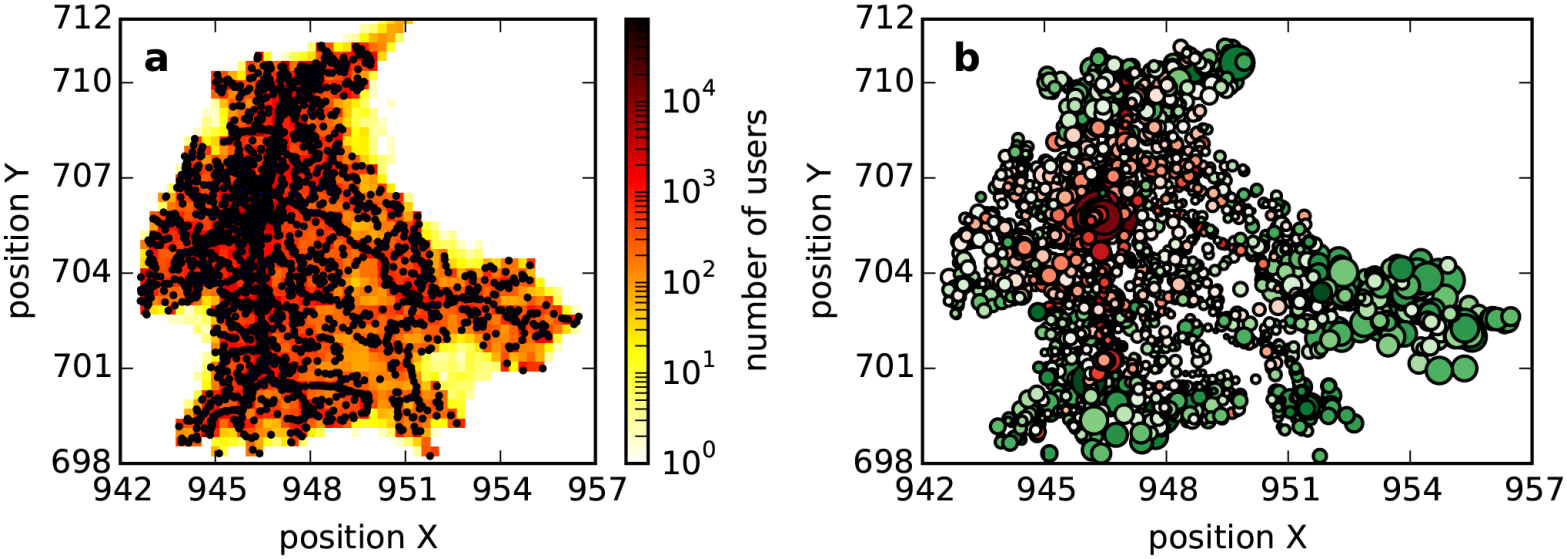
Spatial distribution of service points. (a) is the distribution of human density and position of service points. Nodes denote the service points. We plot the heat map as the background to describe human density; redness is stronger in regions with a large population. Here, a person’s location is approximated by the activity center [26] (defined by ${\vec{r}}_{c}=\frac{1}{n_{u}}\sum_{i=1}^{n_{u}}{\vec{r}}_{i}$, where ${\vec{r}}_{i}$ is the *i*-th service point visited by the person). (b) is the position of two types of nodes. The size of the circle is governed by the clustering coefficient. That is, a larger circle indicates a larger clustering coefficient. The color of the circle is governed by the degree. Low-degree nodes tend to be greener, high-degree nodes tend to be redder, and boundary nodes tend to be whiter.

The other aspect of the spatial influence is the social environment established in the geographical space, which seems more internal. In a human society, people are inclined to live in resource-rich areas. There are more infrastructures such as stores and hospitals in urban areas. In a developing country such as China, the geographical space exhibits an urban-rural dualistic structure. In urban areas, infrastructure is aggregated intensively to serve a large population, while in rural areas, infrastructure is scattered sparsely to serve a small population. In this paper, service points also follow this principle (see Fig 4a). We plot the spatial distribution of two types of nodes in Fig 4b, where red circles represent high-degree nodes and green circles represent low-degree nodes. From this figure, we find that high-degree nodes tend to be located in urban areas, while low-degree nodes tend to be located in rural areas. This observation suggests that due to the distribution of human density and the distance between service points, spatial collaboration patterens differ according to geographic context. This phenomenon is consistent with a city’s urban-rural structure.

**Fig 5:**
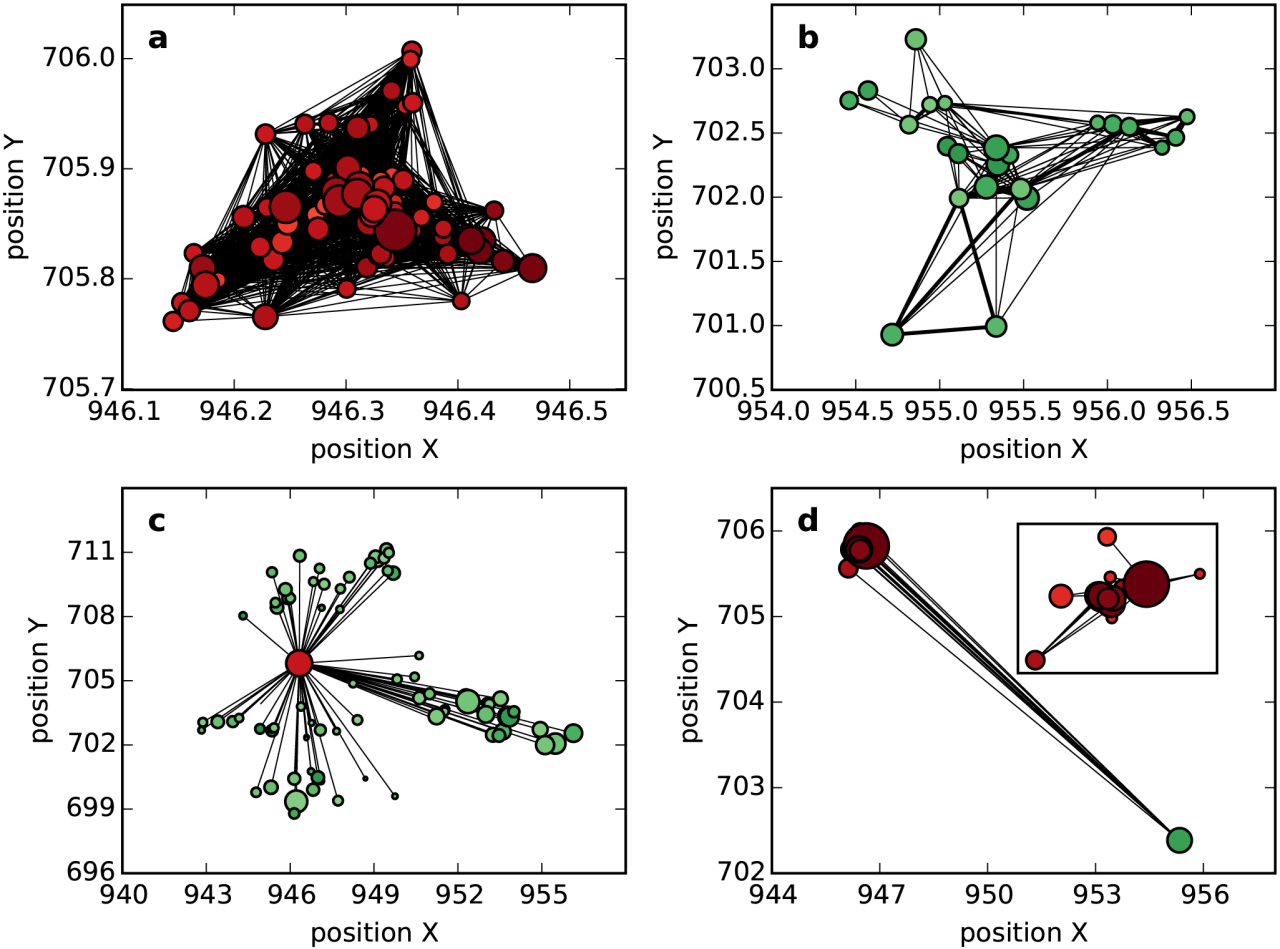
Link pattern of spatially distributed service points. (a) is the link pattern of service points in an urban area. (b) is the link pattern of service points in a rural area. (c) and (d) are the link patterns of service points between urban and rural areas. Here, red circles denote urban service points because they are high-degree nodes, and green circles denote rural service points, because they are low-degree nodes. The width of edges is governed by the weight in the shared network. To simplify the visualization, we drop weak edges with a weight smaller than 0.1.

We further plot the connections between spatially distributed service points in Fig 5. Comparing Fig 5a and 5b, we observe that urban service points forge strong and comprehensive connections with each other, while rural service points are more likely to weakly connect with several nearby others. This result suggests that the densely distributed urban service points are sensitive to small-scale human movement and operate collaboratively to serve local people. They act as high-degree nodes in the shared network, and their intense local structure is consistent with the neighborhood shown in Fig 2a. However, because rural service points are sparsely distributed and serve a smaller population, most of their collaborations are weak or nonexistent. They act as low-degree nodes in the shared network. Their local structure tends to be less intense than that of high-degree nodes, and this is consistent with the neighborhood shown in Fig 2b.

Another factor that influences the network structure is the connections between service points in different types of areas. As shown in Fig 5c and 5d, an urban service point connects with a large number of rural service points. These remote neighbors are at a large distance from each other. A rural service point connects with only a few urban service points, and these remote neighbors are concentrated in a small area. It can be inferred that due to its comprehensive social functions, the urban area receives visitors from different rural areas and that rural people could satisfy their requisitions by visiting a small urban area. Given this skewed social function, urban service points extensively collaborate with numerous rural service points, while rural service points collaborate with only a few urban service points. With such a different link pattern, remote neighbors play different roles. An urban service point's remote neighbors are located far away from each other and exhibit weak inter-collaboration. By contrast, a rural service point's remote neighbors are located within a small area and collaborate well with each other. That is, remote neighbors weaken the local structure intensity for a high-degree node but strengthen it for a low-degree node. Consequently, some high- and low-degree nodes can attain nearly equivalent clustering coefficients in the shared network on the basis that nearby neighbors contribute to a more intense local structure for high-degree nodes than that for low-degree nodes.

Service points located at urban fringes display medium density, which is between that in urban and rural areas. We call them critical service points here. Compared to rural areas, urban fringes receive many rural visitors, and a critical service point is connected with many inter-distant remote neighbors, which negatively impact the local structure intensity. However, because they are located relatively far from each other, as determined by the distance decay effect, critical service points exhibit a weak inter-collaboration. These two aspects result in a low level of local structure intensity for a critical service point, as shown in Fig 4b. This phenomenon characterizes the crisis between high-degree nodes and low-degree nodes. From rural areas to urban fringes, a service point will change from a low-degree node to a critical node and, thus, display a decrease in local structure intensity. From urban fringes to urban areas, a service point will change from a critical node to a high-degree node and, thus, display an increase in local structure intensity. This explains why the clustering coefficient changes inversely with degree for high- and low- degree nodes, as shown in Fig 1b.

The other service points that are helpful in increasing our understanding of the shared network structure are those that approach the urban center. Because the urban center serves the greatest social functions and serves the most people, service points in this area not only collaborate with a large number of other points but also display an extremely dense spatially distribution. Considering the distance decay effect, these service points attain a large number of strong connections. Thus, for an urban service point, as the number of its collaborated neighbors increases, it becomes more likely to be located at the urban center and to attain stronger collaboration. While in the rural areas, which seem to be centerless, it is difficult to find such a pronounced tendency. This is why the interdependency between topology and weights exists only for high-degree nodes, as shown in Fig 1c.

### Communities in the Shared Network

According to the analysis presented above, both high- and low-degree nodes can attain intense local structures, and strong connections prefer to exist between service points that are spatially close to each other. Thus, we can assume that the whole shared network may consist of several spatially separated communities.

**Fig 6:**
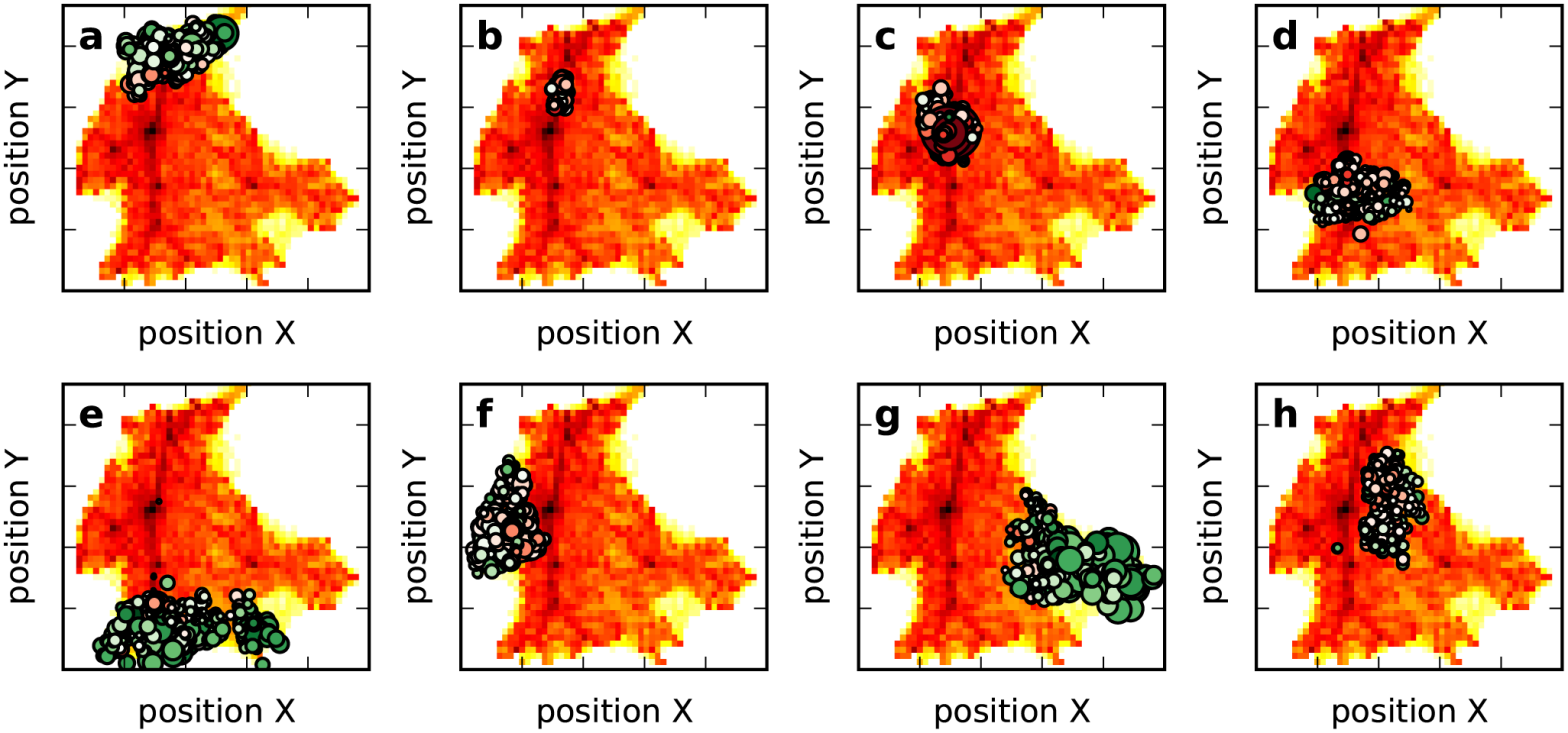
Spatial position of communities in the shared network.

Modularity [27,28] is one of the most popular measures used to identify whether there are community structures in a complex network. It is defined by $Q=\frac{1}{2m}\sum_{i,j}\left[w_{ij}-\frac{s_{i}s_{j}}{2m}\right]\delta (c_{i},c_{j})$, where *c*_*i*_ is the community to which node *i* is assigned, *δ*(*c*_*i*_,*c*_*j*_) is 1 if *c*_*i*_ = *c*_*j*_, and 0 otherwise, *s*_*i*_ is the strength of node *i*, and $m=\frac{1}{2}\sum_{ij}w_{ij}$. Modularity close to 1 indicates that there are significant community structures. For most real-world networks with community structures, the modularity is between 0.2 and 0.7 [28]. We apply a greedy method [29] based on modularity optimization to detect community structures. The result confirms our conjecture that our shared network consists of a number of communities, as the final modularity is as high as 0.61. As shown in Fig 6, these communities are spatially well separated. Given that weak edges dropped in community detection indicate that there are fewer human movements among associated service points, these spatially separated communities practically describe active zones for certain groups of people. Note that communities in urban and rural contexts show different spatial patterns. In urban areas, as shown in Fig 6c, the community is centralized; its service points with intense local structures congregate at the urban center. However, in rural areas, as shown in Fig 6e and 6g, the community is acentric and service points with intense local structures are scattered sparsely.

**Fig 7:**
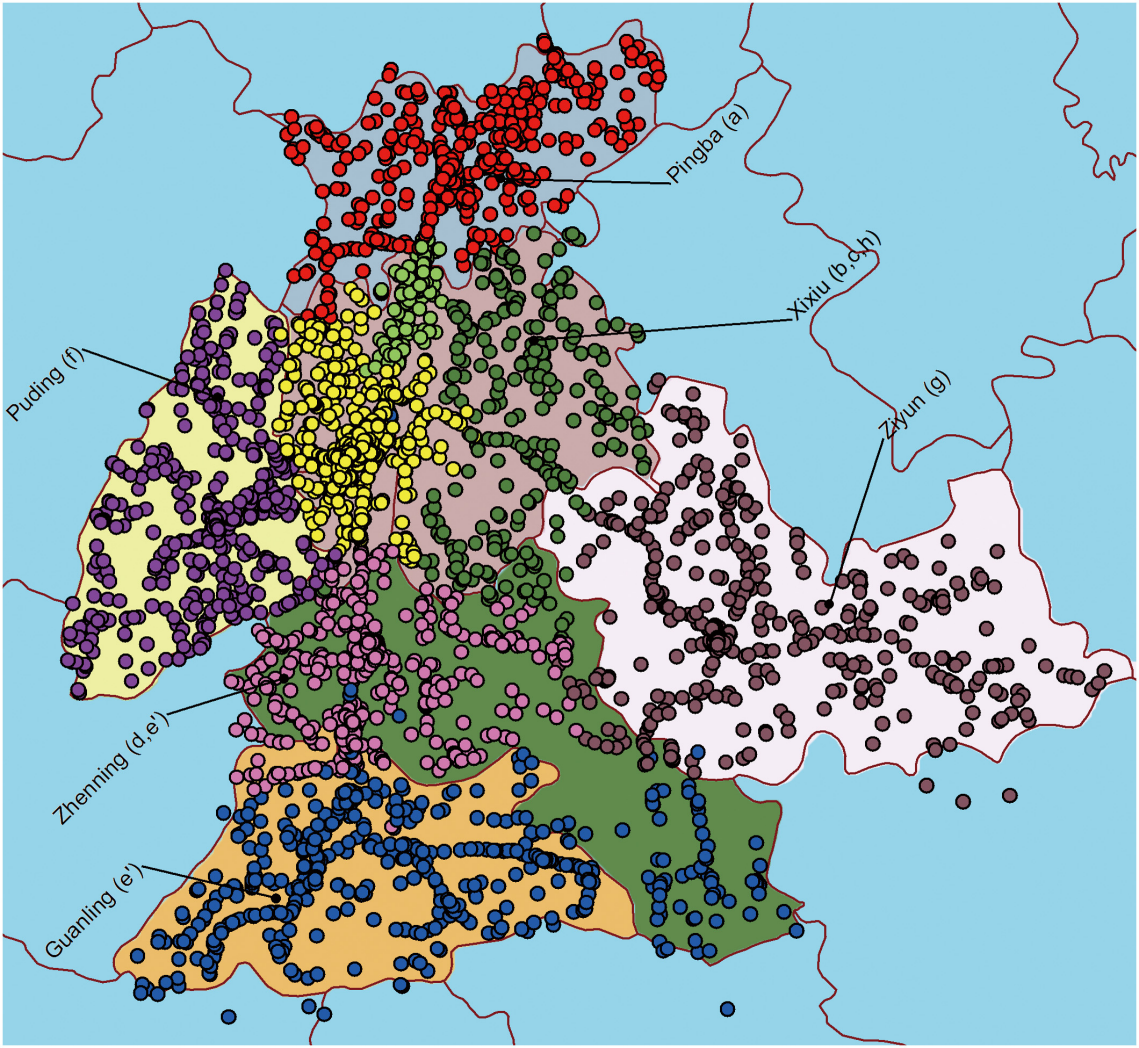
Community vs administrative area. We distinguish communities of infrastructure from the shared network with different colors and use a map of the administrative regions as the background.

Developed based on the cultural and geographical environments, administrative regions are designed by the government to plan the construction of infrastructure in coordination with the local people. However, given the hysteresis of the government's statistic, as well as the complex movement of the population, the administrative region may not always be consistent with the spatial relation driven by human behavior. In our dataset, the city of Anshun consists of six administrative regions, and Xixiu municipal district contains many more residential people than the other five regions. As shown in Fig 7, only half of these regions are consistent with communities from the shared network. The others either include multi-communities or overlap with them. As the municipal district, the region of Xixiu has experienced privileged urban planning for years. Over distributed infrastructures in this region support a more localized collective behavior. Three communities from the shared network are found in this region. Another interesting observation is the regions of Zhenning and Guanling. Although two separate communities are found here, one of them is located in north Zhenning and the other stretches across both Zhenning and Guanling, showing overlap with the administrative regions.

### Incorporating Human Movement to Improve Spatial Clustering

For the effective management of spatially distributed infrastructures, these infrastructures are commonly classified into spatially separated clusters. Although government-designed administrative division results in spatial partition, as discussed above, it may not always match the real spatial property of collective behavior in the city. Another traditional method that seems to be more appropriate is classification of service points according to their resource usage dynamic. However, with a large number of individuals moving and consuming resources at different service points, resource usage at a single service point becomes complicated and the resulting partition from this method may be confused in some cases.

Our analysis of a shared network provides new insight into this problem. A triangular property (shown in Fig 1) distinguishes two types of collaboration patterns exhibited by service points in urban and rural areas. From the global view, an optimized partition of the whole network can assign people to spatially separated regions, which are related to but different from administrative divisions (see Figs 6 and 7). In other words, incorporating human movement may help us divide service points into spatially separated clusters. Here, we incorporate human movement by multiplying the shared network weight with conventional measurement and making the consequent similarity matrix sparse according to the triangular property (see Methods).

**Fig 8:**
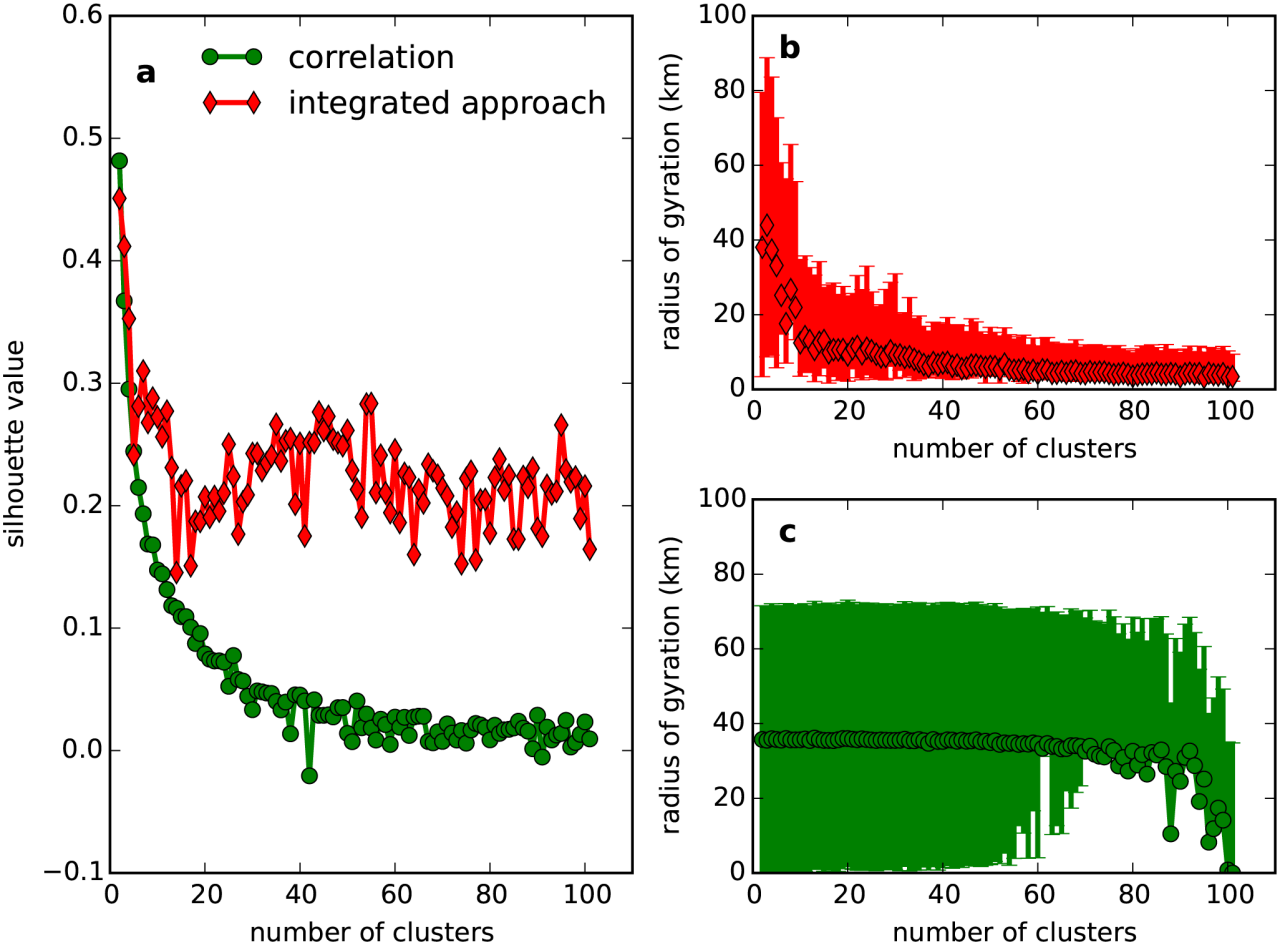
Performance of the clustering. (a) shows the silhouette value at different scales. (b) and (c) show the size of clusters at different scales. We use the radius of gyration [26] to measure the size of the cluster. It is defined as $r_{g}=\sqrt{\frac{1}{n_{c}}\sum_{i=1}^{n_{c}}{\left({\vec{r}}_{i}-{\vec{r}}_{c}\right)}^{2}}$, where ${\vec{r}}_{c}$ is the center of the cluster, ${\vec{r}}_{i}$ is the position of the *i*-th service point, and *n*_*c*_ is the number of service points in the cluster. We plot the semi-quartile and median of *r*_*g*_ in the *y*-axis to study the change in the cluster's spatial dispersion.

**Fig 9:**
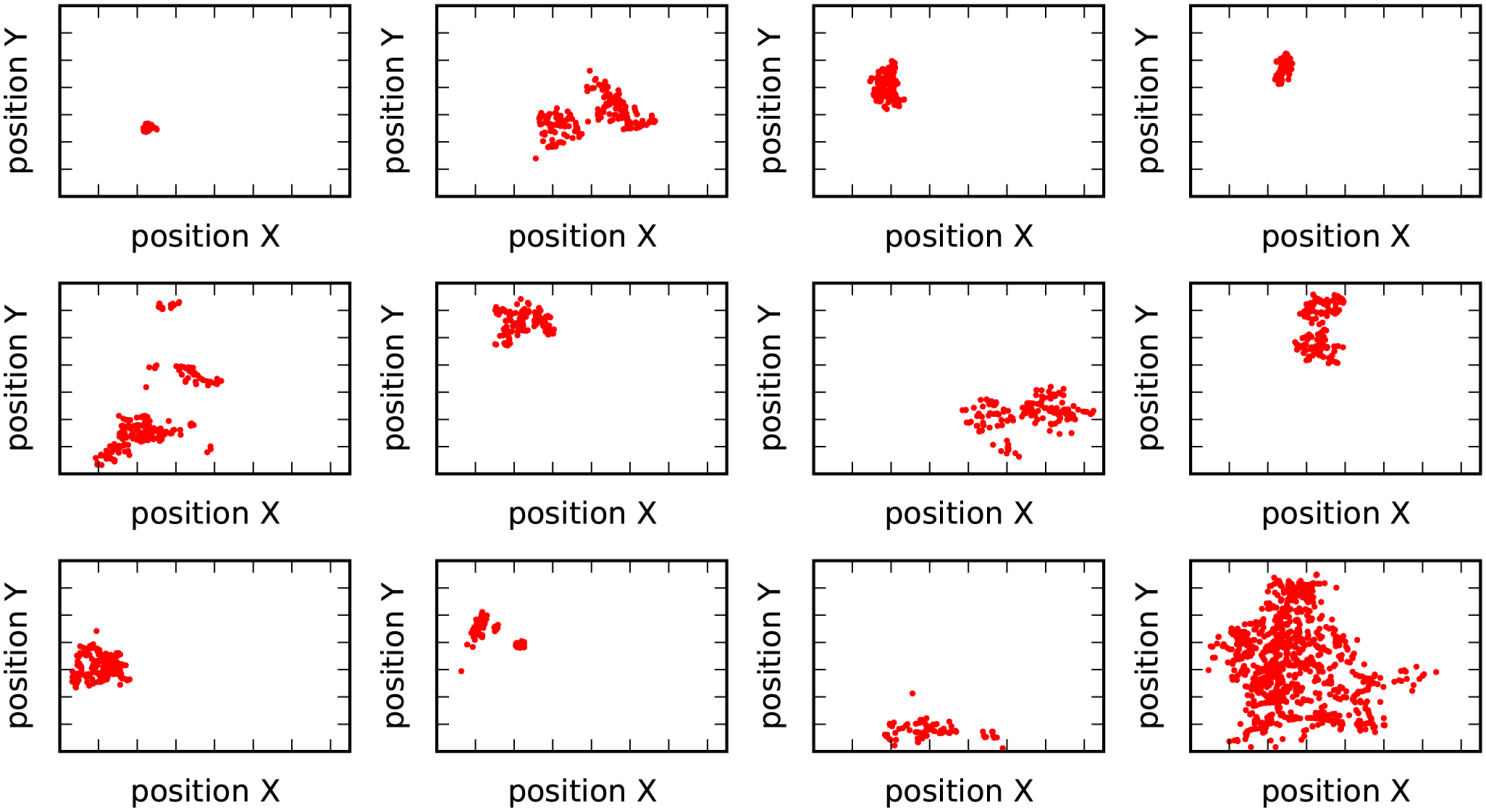
Spatial distribution of clusters resulting from the proposed method.

**Fig 10:**
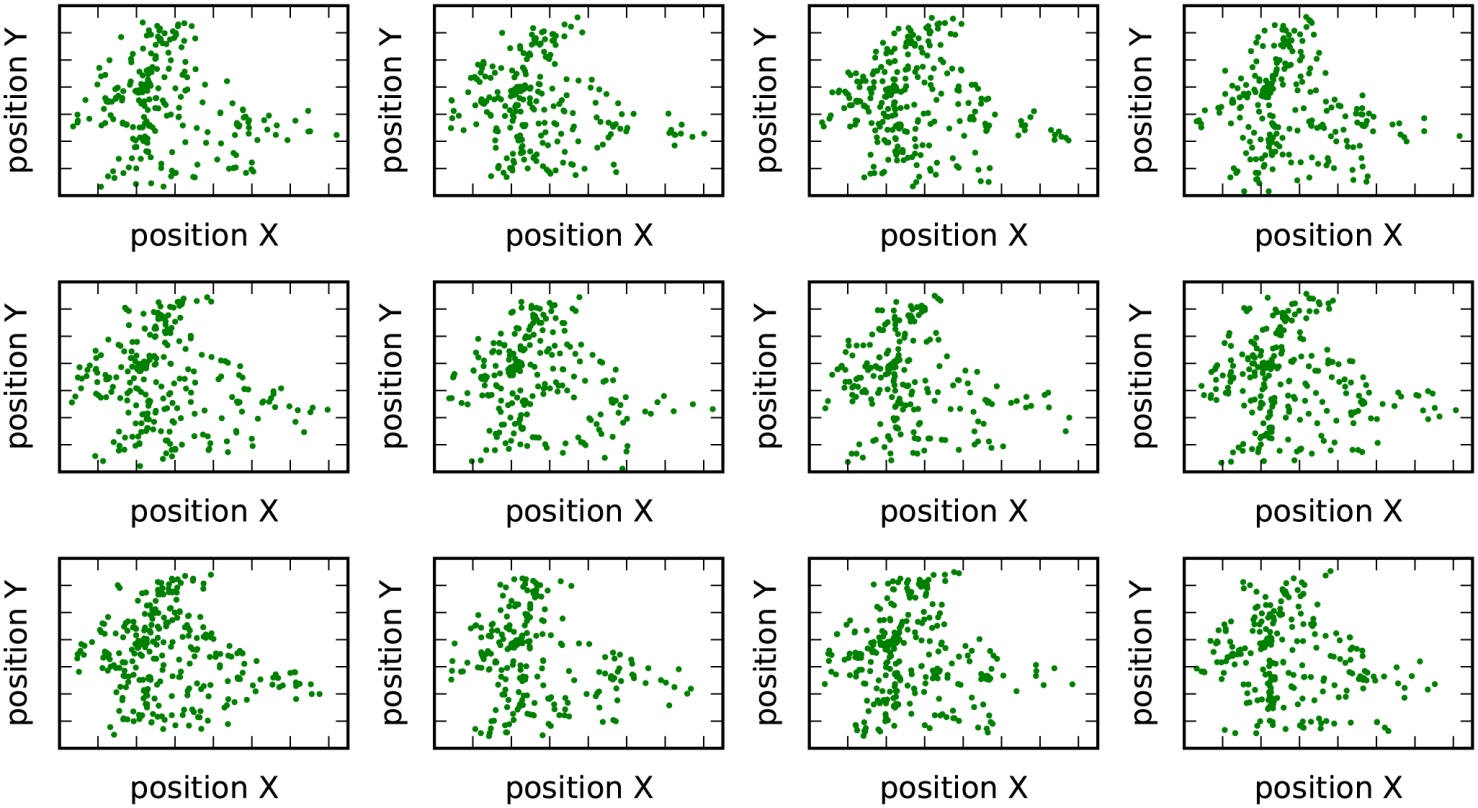
Spatial distribution of clusters resulting from the conventional method.

As shown in Fig 8a, the proposed method results in a larger and more fluctuated silhouette value [30] than conventional method, leading to well-defined clusters in the feature space. In the geographical space, most clusters from our proposed method are located in spatially separated small regions (see Fig 9). Furthermore, as shown in Fig 8b, the radius of gyration of these clusters shrinks rapidly as the number of clusters increases and finally converges to a similar small value. This indicates that the very large cluster in Fig 9, which seems to be negative, will be further divided into smaller clusters when clustering is performed at a more detailed scale. However, with the conventional method, clusters overlap with each other in the space (see Fig 10). The almost constant radius of gyration (see Fig 8c) indicates that overlap will occur at all clustering scales.

These results confirm the outperformance of the proposed method. In some cases, such as in our dataset, the complicated external resource consumption dynamic may fail to classify spatially distributed infrastructure. Because it considers the spatial relation derived by human movement, our proposed method can obtain well-defined clusters. Service points in the same cluster not only experience a similar external resource dynamic, but also collectively constitute local people’s active zone, i.e., the zone where most of their resource consumption behaviors occur. These clusters are spatially separated from each other, dividing a large area into a number of smaller regions. For each cluster, the resource consumption patterns of service points are similar and the total required resources can be estimated easily, leading to more effective resource management.

## Conclusion

In this paper, using the empirical data from a cellular network in one city in western China, we construct a shared network to study the collaboration of spatially distributed infrastructures, which we treat as service points. Our findings reveal some interesting spatial structures in human society on the intra-city scale. This network is not scale-free; rather, it exhibits a triangular property governed by two types of nodes with different link patterns, which are compatible with the urban-rural dualistic structure of the city. The connections in the shared network display the basic distance decay effect. In urban areas, where infrastructures are densely distributed and perform an extensive social function, service points not only form comprehensive and strong collaborations with each other, but also collaborate with many spatially dispersed rural service points, which are rarely inter-collaborative and weaken the intensive local structure. While in rural areas, where infrastructures are distributed sparsely, service points collaborate with only some nearby neighbors, forming a sparse local structure. However, because the remote urban service points are spatially close to each other, their collaboration plays a positive role in determining local structure intensity. Thus, both of these types of nodes in the shared network can ultimately reach the same level of local structure intensity. We further find that the network consists of many communities. Urban communities are centralized as their service points with intense local structure congregate at urban centers. By contrast, rural communities are decentralized, as their service points with intense local structure scatter sparsely. These communities are spatially separated from each other, characterizing the active zones of local people. However, only half of them completely overlap with the administrative regions designed by governments.

Another contribution of our work lies in the creation of a new approach for infrastructure clustering. Classifying spatially distributed infrastructure into small clusters is an effective way to simplify management. However, by considering only the external resource usage dynamic, and dropping internal human behavior, the conventional method may not always sufficiently distinguish different groups of service points, such as those in our dataset. Enlightened by the observations of the shared network, we propose the incorporation of human movement behavior into infrastructure clustering. Our new approach results in improvements in both features and geographical spaces. The well-defined spatially separated clusters not only distinguish service points with different resource consumption patterns, but also make it possible to obtain a more refined estimate of the spatial resource requirement.

Finally and as an extension, the shared network projects the collective human behavior on the spatially distributed service points. It presents a new perspective to better understand the spatially distributed resource supply system while considering consumers' movement. As an important part of life of many species, movement behavior may have a significant impact on spatial resource consumption for phenomena such as animal migration. The shared network approach can also be used in these fields to further explore the spatial structure of the ecosystem.

## Methods

### Data Description

A dataset based on UDRs was created in Anshun, China. It contains data access records of 1.2 million mobile phone users in 20 days, covering 4,000 base stations. The city of Anshun is located in a remote area of western China and has a typical urban-rural dualistic structure in regards to spatial economic development. In this city, urban areas contain many more people and infrastructures than rural areas. Compared with previous studies based on CDRs [26, 31, 32], taking advantage of the UDRs, we extend the tracking of human movement from mobile call behavior to data usage behavior. Furthermore, wireless communication resource usage is assessed via traffic load. Moreover, given that data usage behaviors are more common in our daily lives, UDRs provide a more detailed description of human movement. In our dataset, a UDR contains the fields shown in Table 1. It records the detailed quantity of the resource consumed at the service point.

**Table 1:** Description of UDR fields.

Fields	Description
UID	An encrypted telephone number indicating a mobile user.
Time_start	The time that a user begins the data usage.
Time_end	The time that a user ends the data usage.
Lac & cid	The base station providing wireless communication resources, which acts as a service point.
Load_out	The traffic load used by the user, which quantifies resources consumed by the user.

### Construction of the Shared Network

**Fig 11:**
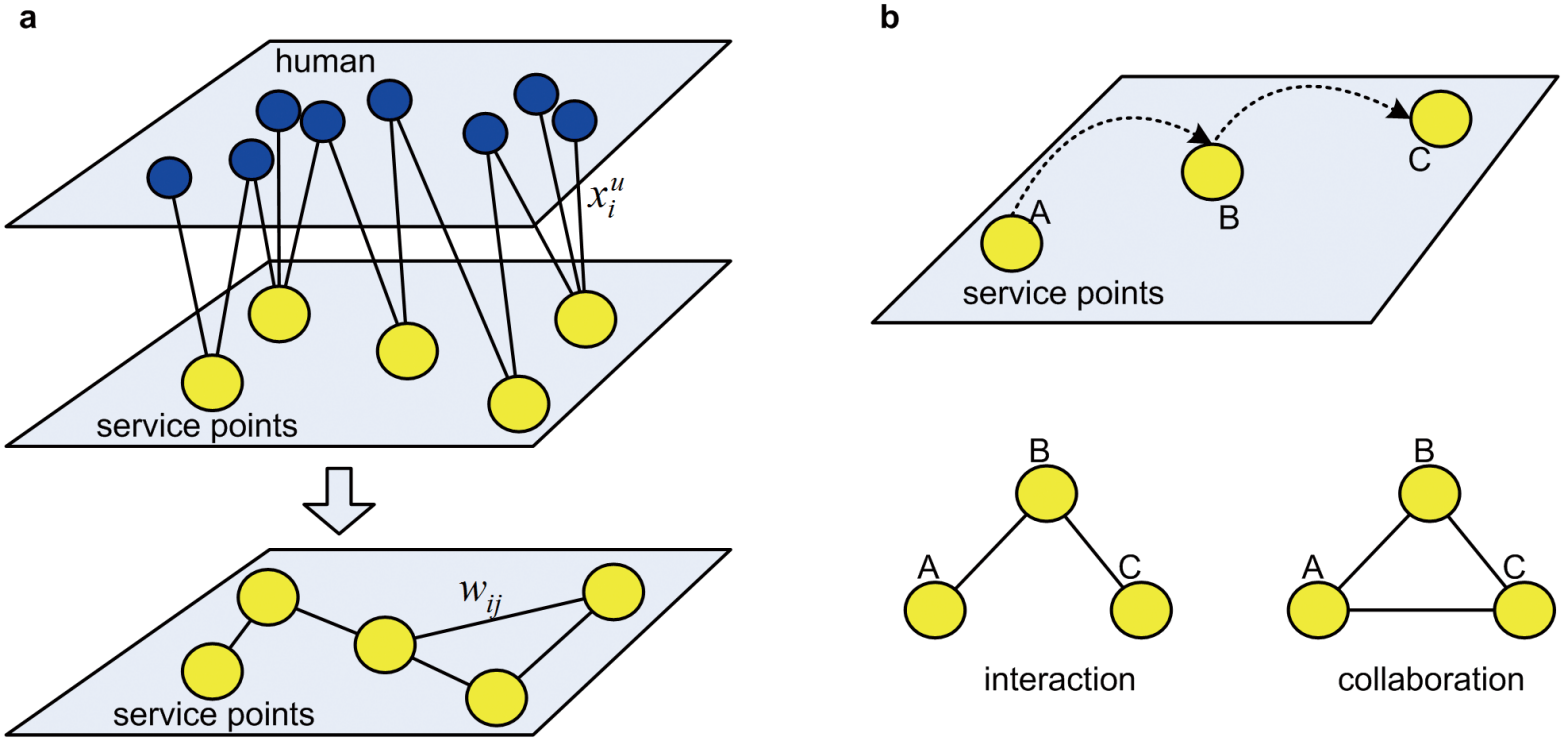
Construction of the shared network. (a) shows the procedure for constructing the shared network. Blue nodes represent humans, and yellow nodes represent service points. (b) shows the difference between the concept of interaction and collaboration in the network construction.

Base stations in a cellular network act as service points that provide wireless communication resources. We can use a bipartite graph to represent the relationship between base stations and human users, as shown in Fig 11a. Considering people’s daily movement between home and workplaces, each person consumes resources at a number of service points. The edge with a weight $x_{i}^{u}$ here characterizes the resource that service point *i* provides to person *u*. Because they serve mobile users, service points operate collaboratively and function as a spatially distributed resource supply system. To study the collaboration among these service points, we propose to construct a shared network according to people's resource consumption behavior, which can be viewed as a transformation of the bipartite network.

In the shared network, the node represents the service point, and the weighted edge measures the collaboration between service points formed in the process of providing resources to the same group of people. If two service points are visited by the same person, there will be an edge between them in the shared network (see Fig 11a). The weight of the edge is defined as

$$w_{ij}=\sum_{u}\frac{\delta_{i}^{u}\delta_{j}^{u}}{n_{u}-1}\times \frac{x_{i}^{u}+x_{j}^{u}}{X^{u}}$$ (2)

where $\delta_{i}^{u}$ is 1 if the service point *i* is visited by the person *u*, or 0 otherwise. $x_{i}^{u}$ is the quantity of the resource provided by service point *i*. *n*_*u*_ is the number of service points serving the person *u*. *X*^*u*^ is the total resource consumed by the person *u*. To characterize the accumulated effects of the spatial environment on collective human behavior, we do not consider a time window in this procedure. That is, if a person visits the first service points, whenever he visits another service point later in our data set, there will be a connection added between these two service points in the shared network. Note that our shared network is different from the interaction network given in [18, 33], which is widely used in transportation scenarios. In an interaction network, edges connect nodes according to the trajectory of human movement and the weight edge is used to measure the exchange of people between spatial units. However, the shared network is more likely to be a collaboration network. It adopts the concept of the scientist collaboration network [27] but has the physical meaning that nodes contain spatial information, and edges are generated by integrating human movement and resource usage behavior. We determine the difference between the concept of interaction and collaboration in the network construction in Fig 11b. If a person visits service points A, B and C successively, there will be two edges (A, B) and (B, C) in the interaction network. However, in our shared network, because all three service points are visited by the same user, there will be another edge (A, C).

### Incorporating Human Movement into Spatial Clustering

Adopting the view of external resource consumption, conventional methods typically use some time series metrics to cluster spatially distributed service points. In a cellular network, the Pearson's correlation of traffic dynamics has been used to measure the similarity between base stations [34]. Given the movement of a large number of users, as well as different resource usage practices, the resulting resource consumption dynamic on service points may not always effectively classify them. According to the community structure in the shared network, by dropping the weak collaborations among service points, we can divide infrastructure into spatially separated clusters, each of which can be viewed as a separate subsystem serving a certain group of people. The incorporation of human movement into the clustering of service points may improve the result in situations where conventional methods do not work well.

We use the weighted edge in the shared network to measure the closeness of the relationship between service points, as determined by mobile users' resource consumption. Furthermore, we integrate the weighted edge with the conventional time series metric to measure the similarity between service points. Thus, service points in the same cluster not only display a similar resource consumption dynamic, but also serve the same group of people. Because resource usage behavior is correlated with personal features [32], this measurement may lead to a more stable collective performance of resource consumption for each cluster. Moreover, because the size of neighborhoods with an intense local structure is larger for urban service points and smaller for rural service points, we can make the boundaries of clusters sharper if we drop different numbers of neighbors for these two types of nodes in the similarity matrix. Thus, taking advantage of the triangular distribution of the degree in the shared network, we can further improve clustering by tranfroming the similarity matrix into a sparse matrix. In particular, we employ a new clustering approach as follows.

#### Similarity Definition

First, we define a new similarity as

$$a_{ij}=\frac{r_{ij}-r_{\text{min}}}{r_{\text{max}}-r_{\text{min}}}\times \frac{w_{ij}-w_{\text{min}}}{w_{\text{max}}-w_{\text{min}}}$$ (3)

where *w*_*ij*_ is the edge weight between service points *i* and *j* in the shared network, which is given by Eq (2). The fraction $\frac{w_{ij}-w_{\text{min}}}{w_{\text{max}}-w_{\text{min}}}$ behind the multiplier is the normalized pattern. $a_{ij}^{\prime }=\frac{r_{ij}-r_{\text{min}}}{r_{\text{max}}-r_{\text{min}}}$ can be viewed as the normalized Pearson's correlation *r*_*ij*_ of the resource consumption time series, which is widely used in conventional methods.

#### Sparse Similarity Matrix

We then transform the consequent similarity matrix *A*_0_ = {*a*_*ij*_} into a sparse matrix, where *a*_*ij*_ is defined by Eq (3). According to the above analysis, in the shared network, the high-degree node with more neighbors has an intense local structure, while the opposite is true for the low-degree node. Thus, retaining more neighbors for high-degree nodes but fewer neighbors for low-degree nodes in the transformation, we can obtain sharper clusters. Considering the triangular distribution of the degree, we retain *n*_*i*_ neighbors for service point *i*, which is given by

$$n_{i}=\{\begin{array}{cc} n_{\text{max}}-\frac{n_{\text{max}}-n_{\text{min}}}{m-l}\times \left(k_{i}-l\right) & k_{i}<=m,\\ n_{\text{max}}-\frac{n_{\text{max}}-n_{\text{min}}}{r-m}\times \left(r-k_{i}\right) & k_{i}>m. \end{array}$$ (4)

where *k*_*i*_ is the degree of service point *i* in the shared network. *l*, *m* and *r* are parameters of the triangular distribution in Eq (1). The other two parameters *n*_min_ and *n*_max_ are, respectively, the minimum and maximum number of neighbors we set for the whole data.

#### Spectral Clustering

Finally, we implement a spectral clustering [35, 36] algorithm to partition the sparse similarity matrix to produce a classification of service points. The spectral clustering method used herein is summarized as follows. Let *A* = {*a*_*ij*_} be the sparse similarity matrix, we

- compute the Laplacian matrix *L* = *I*-*D*^-1/2^*AD*^-1/2^, where *D* is the diagonal matrix with the $d_{i}=\sum_{j=1}^{j=n}a_{ij}$ on the diagonal, and *I* is the identity matrix;
- compute the first *k* eigenvectors *e*_1_,…,*e*_*k*_ of *L*;
- let *V*∈ℝ^*n*×*k*^ be the matrix containing the vectors *e*_1_,…,*e*_*k*_ as columns, form the matrix *T*∈ℝ^*n*×*k*^ from *V* by setting $t_{ij}={e_{ij}/\left(\sum_{k}e_{ik}^{2}\right)}^{1/2}$; and
- for *i* = 1,…*n*, let *y*_*i*_∈ℝ^*k*^ be the vector corresponding to the *i*-th row of *T*, (*y*_*i*_)*i* = 1,…,*n* are clustered into clusters *C*_1_,…,*C*_*k*_ using *k*-means algorithm.

For the sake of comparison, we use the conventional method to classify service points. In this comparison, without considering human movement, we define similarity between service points using the normalized Pearson's correlation $a_{ij}^{\prime }=\frac{r_{ij}-r_{\text{min}}}{r_{\text{max}}-r_{\text{min}}}$ and then retain the same *n*_0_ neighbors for every service point. We also use the spectral clustering algorithm to partition the resulting sparse similarity matrix.
